# Which patients with para-aortic lymph node (LN16) metastasis will truly benefit from curative pancreaticoduodenectomy for pancreatic head cancer?

**DOI:** 10.18632/oncotarget.8690

**Published:** 2016-04-11

**Authors:** Chen Liu, Yu Lu, Guopei Luo, He Cheng, Meng Guo, Zuqiang Liu, Jin Xu, Jiang Long, Liang Liu, Deliang Fu, Quanxing Ni, Min Li, Xianjun Yu

**Affiliations:** ^1^ Department of Pancreatic and Hepatobiliary Surgery, Fudan University Shanghai Cancer Center, Shanghai, P.R. China; ^2^ Department of Oncology, Shanghai Medical College, Fudan University, Shanghai, P.R. China; ^3^ Pancreatic Cancer Institute, Fudan University, Shanghai, P.R. China; ^4^ Department of Pancreatic Surgery, Huashan Hospital, Shanghai Medical College, Fudan University, Shanghai, P.R. China; ^5^ Department of Medicine, Department of Surgery, The University of Oklahoma Health Sciences Center, Stanton L. Young Biomedical Research Center, Oklahoma City, USA

**Keywords:** pancreatic cancer, para-aortic lymph node, lymphadenectomy, metastasis, prognosis

## Abstract

In patients with cancer of the pancreatic head, metastasis to para-aortic lymph nodes (LN16) is considered distant metastasis and a poor prognostic marker. However, the incidence of LN16 involvement in pancreatic head cancer is high, and it is unclear whether all such patients have poor surgical outcomes. We investigated the significance of LN16 involvement in resectable pancreatic head cancer by retrospectively analyzing 579 ductal adenocarcinoma patients treated with para-aortic lymph node dissection at two high-volume Chinese centers. Depending upon tumor location, the incidence of LN16 metastasis and the correlation between LN16 involvement and involvement of Group 1 or 2 lymph nodes significantly differed. Metastasis to LN16 indicated a high serum tumor burden and a poor prognosis, though LN16-positive patients with a lymph node ratio (LNR) < 0.25 may still benefit from radical surgery. Survival analysis of LN16-positive patients with resectable pancreatic head cancer revealed that tumor size, tumor differentiation, and tumor location are independent prognostic factors. We also found that preoperative serum CA125 < 18.62 U/ml and the level of JAK2 signaling are both indicators of who may benefit from curative surgical resection for pancreatic head cancer.

## INTRODUCTION

The incidence of pancreatic adenocarcinoma, which has an average five-year survival rate of about 6%, has increased in China over the past several decades [1]. Among patients diagnosed with pancreatic cancer, less than 20% are eligible for a curative resection [2]. Consequently, it is extremely important to conduct an optimal surgery and extensive lymphadenectomy during surgery for every resectable case of pancreatic cancer.

The status of lymph node involvement is a critical prognostic factor for pancreatic ductal adenocarcinoma [3]. However, there is little agreement regarding what constitutes an optimal lymphadenectomy during pancreatic resection. In most randomized controlled trials (RCTs) or authoritative consensus statements, a standard lymphadenectomy including Group 1 and partial Group 2 lymph node stations, such as lymph nodes (LN) 5, 6, 8a, 12b, 12c, 13, 14a, 14b, and 17, is recommended during pancreaticoduodenectomy. Extended resection including partial Group 2 and Group 3 lymph node stations, such as LN 8p, 9, 12a, 12p, 14c, 14d, 15, and 16, should not be routinely performed, due to lack of evidence that the patients will actually benefit from this high-risk surgery [4, 5].

Despite being classified as Group 3 lymph nodes, para-aortic lymph node (LN16) involvement is commonly detected among patients with resectable pancreatic head carcinoma, ranging from 18.4% to 26% [6–8]. The necessity of LN16 resection is still controversial. Several clinical studies have indicated that metastasis to LN16 implies systemic metastasis, and the resection is therefore not recommended. Another study indicated that some cohorts of LN16-positive patients might actually benefit from extended resection [9–11]. Sakai et al. investigated the lymph node metastatic pattern for pancreatic head cancer, and found that the metastasis to para-aortic lymph nodes was always accompanied by involvement of LN13, 14, and 17, which were “junctional lymph nodes “flowing to LN16 [12]. However, another study from a different center reported that there was no significant correlation between LN16 and any other lymph node station, except for LN12 [13].

In this study, we retrospectively analyzed patients with para-aortic lymph node dissection during pancreaticoduodenectomy for ductal adenocarcinoma of the pancreatic head in two high-volume Chinese centers. We sought to determine whether LN16 should be classified into Group 3 lymph node station in every case, and what metastasis to LN16 actually meant for resectable pancreatic head cancer. Our results indicate that a subgroup of patients might benefit from a curative surgery, even with involved para-aortic lymph nodes.

## RESULTS

### LN16 should be classified based on the tumor location of pancreatic head cancer

**Figure 1:**
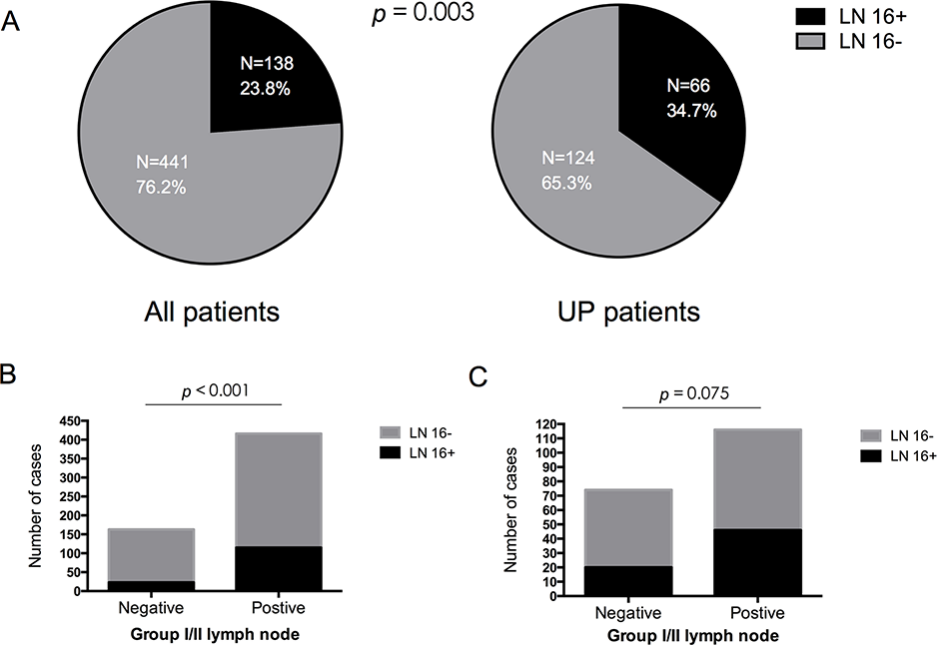
(**A**) The incidence of para-aortic lymph node metastasis based on tumor location in pancreatic head cancer. In the entire sample (*N* = 579), para-aortic lymph node metastases were found in 138 patients (23.8%), while the incidence of LN16 involvement increased to 34.7% (66/190) of patients with tumors located in the uncinate process of the pancreas (UP; *p* = 0.003). (**B**–**C**): Correlation between LN16 status and Group I/II lymph nodes status in patients with resected pancreatic head cancer. LN16 status was associated with Group I/II lymph node status in all patients (B) *p* < 0.001), while no significant relationship could be found between LN16 status and Group I/II lymph node status in patients with tumors located in the uncinate process of the pancreas (C) (*p* = 0.075).

We categorized 579 patients with resected pancreatic head cancer by tumor location: 190 patients had dorsal pancreatic head cancer (tumor located in uncinate process) and 389 patients had tumors in other locations. In the entire sample, LN16 metastases were found in 138 patients (23.8%). However, when the search was restricted to dorsal pancreas cases, the incidence of LN16 involvement increased to 34.7% (66/190; *p* = 0.003, Table 1, Figure 1). Furthermore, although there was a strong correlation between metastasis to LN16 and to lymph nodes Group 1 or 2 in the entire sample (*p* < 0.001), the significance unexpectedly disappeared when we examined dorsal pancreatic tumors as a unique subgroup (*p* = 0.075, Table 1, Figure 1).

**Table 1:** Clinical and pathologic findings in pancreatic head cancer patients with or without LN16 metastasis

Variables	LN16 positive (*N* = 138)	LN16 negative (*N* = 441)	*P*
Age, mean ± SD, year	61.93 ± 9.64	61.21 ± 10.16	0.464
Sex, F/M	50/88	193/248	0.118
Preoperative CA19-9, mean ± SD, U/ml	594.40 ± 1313	484.00 ± 1729	0.038*
Tumor Location (UP/Other)	66/72	124/317	< 0.001
Differentiation (Well/Poor)	63/75	269/172	0.002
Tumor size, mean ± SD, cm	3.39 ± 1.44	3.32 ± 1.40	0.787*
Neural infiltration (No/Yes)	57/81	180/261	0.919
Portal vein invasion (No/Yes)	67/71	233/208	0.379
Vascular emboli (No/Yes)	75/63	230/211	0.652
ELN, mean ± SD	18.80 ± 12.25	17.49 ± 12.00	0.207*
PLN, mean ± SD	3.64 ± 3.24	2.47 ± 3.06	< 0.001*
LNR, mean ± SD	0.30 ± 0.28	0.21 ± 0.39	< 0.001*
Group I/II lymph nodes involved (No/Yes)	23/115	140/301	< 0.001
Adjuvant treatment (No/Yes)	9/129	42/399	0.278

LN indicates lymph node; CA19-9, carbohydrate antigen 19–9; UP, uncinate process of pancreas;

* Mann-Whitney test; ELN, Examined lymph nodes; PLN, Positive lymph nodes; LNR, Lymph node ratio.

### Metastasis to LN16 in pancreatic head cancer indicates high tumor burden and overall poor prognosis

Table 1 shows the clinicopathological variables in pancreatic head cancer patients with (*N* = 138) and without LN16 metastasis (*N* = 441). LN16-positive patients had higher positive lymph nodes (PLN; *p* < 0.001), higher lymph node ratio (LNR; *p* < 0.001), and higher preoperative serum CA19-9 levels (594.4 vs. 484.0 U/ml, *p* = 0.038) compared to LN16-negative patients.

**Figure 2:**
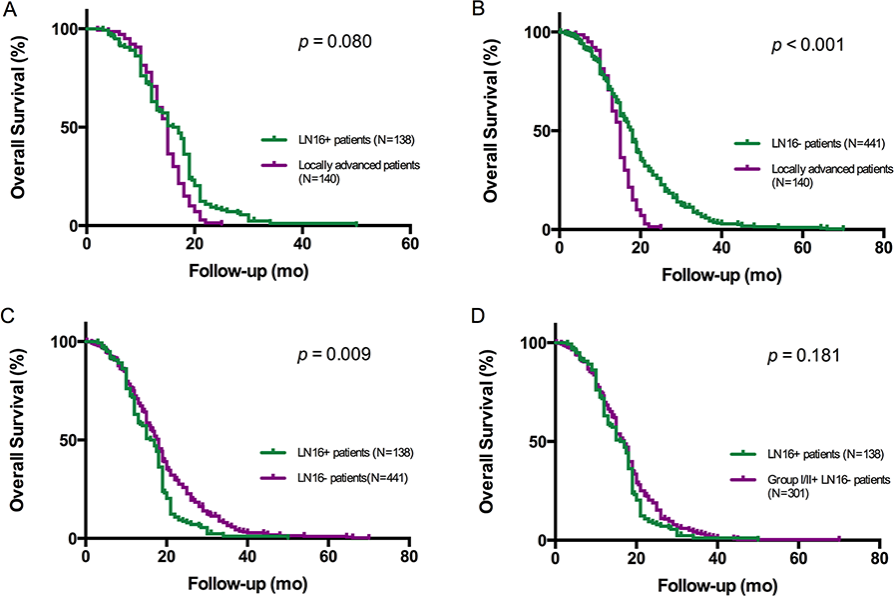
Kaplan-Meier analysis of overall survival in patients with pancreatic head cancer The survival of patients with unresectable, locally advanced tumors was significantly shorter than that of patients with surgically resected disease without LN16 involvement **(B)** (*p* < 0.001), but close to that of patients with LN16 metastasis who underwent curative surgery **(A)** (*p* = 0.080). In the resected group, the survival of patients with LN16 metastasis was significantly shorter than that of LN16-negative patients **(C)** (*p* = 0.009), but there was no difference in overall survival between LN16-positive patients and LN16-negative patients with positive Group I/II lymph nodes **(D)** (*p* = 0.181).

Kaplan-Meier survival analysis indicated that overall survival of 138 resected pancreatic cancer patients with positive LN16 was similar to that of 140 patients with unresectable, locally advanced disease (*p* = 0.080, Figure 2). The overall survival of LN16-positive patients was significantly worse than that of LN16-negative patients (*p* = 0.009, Figure 2). However, the survival of the patients with negative LN16, but positive Group 1 or 2 lymph nodes, was not better than that of LN16-positive patients (*p* = 0.181, Figure 2).

### Univariate and multivariate survival analysis of prognostic factors for LN16-positive patients with resectable pancreatic head cancer

LN16-positive pancreatic head cancer patients had a median survival of 16.5 months. Ten clinicopathological factors were investigated to determine their prognostic significance in resectable pancreatic cancer with LN16 metastasis. Univariate analysis revealed that poor tumor differentiation, primary tumor size < 3 cm, and a non-uncinate process tumor location were associated with poor survival in LN16-positive patients. Multivariate survival analysis identified that tumor differentiation, tumor size and tumor location were three independent prognostic factors for the LN16-positive population (Table 2).

**Table 2:** Univariate and multivariate survival analysis of prognostic factors for pancreatic head cancer patients with positive LN16

	Hazard ratio	95% CI	*P*
**Univariate analysis**			
Age (< 63/≥ 63, year)	1.254	0.882–1.764	0.193
Sex (F/M)	1.289	0.906–1.833	0.158
Preoperative CA19-9(< 37/≥ 37, U/ml)	1.022	0.688–1.518	0.914
Differentiation (Poor/Well)	1.587	1.122–2.244	0.009
Tumor Location (Other/UP)	1.727	1.206–2.474	0.003
Tumor size (< 3/≥ 3, cm)	1.513	1.065–2.150	0.021
Neural infiltration (No/Yes)	0.838	0.593–1.184	0.317
Vascular emboli (No/Yes)	0.887	0.630–1.248	0.490
Portal vein invasion (No/Yes)	0.862	0.614–1.208	0.388
Adjuvant treatment (No/Yes)	1.240	0.628–2.449	0.536
**Multivariate analysis**			
Differentiation (Poor/Well)	1.477	1.039–2.100	0.030
Tumor Location (Other/UP)	1.614	1.122–2.231	0.010
Tumor size (< 3/≥ 3, cm)	1.471	1.034–2.093	0.032

CI indicates confidence interval; UP, uncinate process of pancreas.

### LNR is associated with overall survival of pancreatic head cancer patients with LN16 metastasis

**Figure 3:**
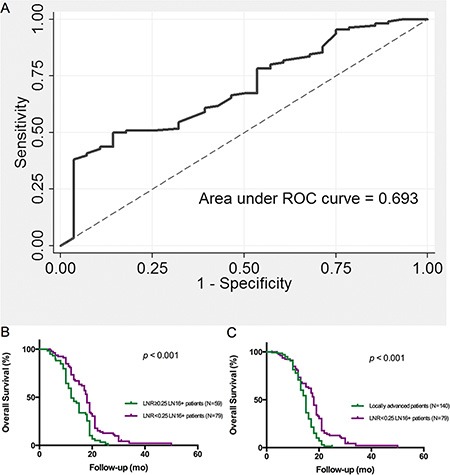
Lymph node ratio (LNR) is associated with overall survival of pancreatic head cancer patients with LN16 metastasis (**A**) ROC analysis of LNR indicating the survival of pancreatic cancer patients with LN16 metastasis. The optimal cut off vale is 0.25. The ROC area of LNR is 0.693 with a sensitivity of 50.0% and a specificity of 85.7%. (**B**) The survival of LN16-positive patients with LNR < 0.25 was longer than patients with LNR ≥ 0.25 (*p* < 0.001). (**C**) The survival of LN16-positive patients with LNR < 0.25 was longer than that of locally advanced cases (*p* < 0.001).

Previously, we found that pancreatic cancer patients with higher LNR experienced a poorer surgical outcome [14]. In this study, LNR was also evaluated as a prognostic factor in 138 LN16-positive patients with pancreatic head cancer. Receiver operating characteristic (ROC) analysis was used to determine the cut-off value in patients with LN16-involved pancreatic head cancer after radical surgery. The area under ROC curve was 0.693, with a sensitivity and specificity of 50% and 85.71% respectively, when using LNR = 0.25 as a cut off value. Kaplan-Meier analysis found that overall survival of patients with LNR < 0.25 was significantly longer than that of patients with LNR ≥ 0.25 in LN16-positive cohort (*p* < 0.001, Figure 3). The survival of LN16-positive patients with LNR < 0.25 was also longer than that of locally advanced cases (*p* < 0.001, Figure 3).

### Preoperative serum CA125 level predicts the prognosis of patients with pancreatic head cancer with LN16 metastasis

**Figure 4:**
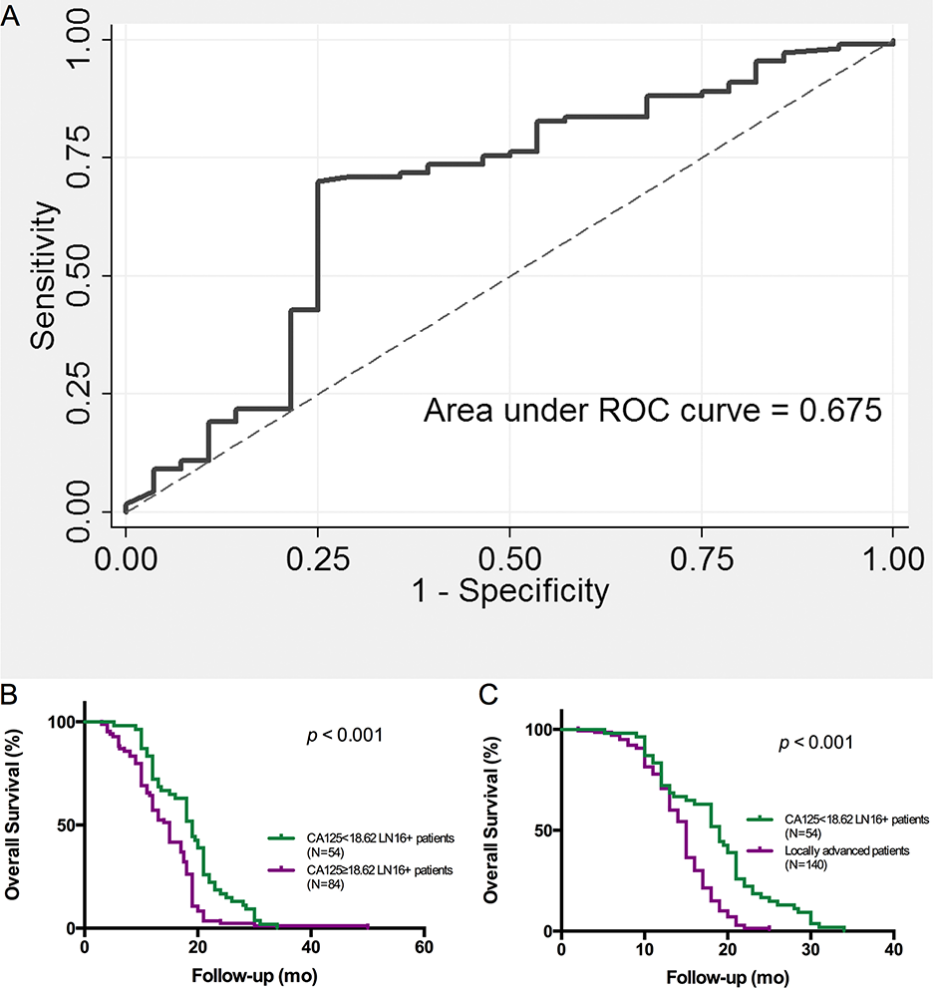
Preoperative serum CA125 level indicates the surgical outcomes of patients with pancreatic head cancer with LN16 metastasis (**A**) ROC analysis of CA125 in predicting the survival of LN16-positive patients. The area under ROC curve is 0.675. The sensitivity and specificity were 70.0% and 75.0% respectively, at a cutoff value of 18.62 U/ml. (**B**) The survival of patients with preoperative CA125 level < 18.62 U/ml was longer than that of patients with preoperative CA125 ≥ 18.62 U/ml (*p* < 0.001). (**C**) The patients with preoperative CA125 level < 18.62 U/ml benefit from surgery, even with para-aortic lymph node metastasis (*p* < 0.001).

As reported previously, the gynecological tumor-associated biomarker CA125, also known as mucin-16, is superior to CA19-9 in predicting the resectability of pancreatic cancer [15]. More importantly, high serum CA125 levels often indicate metastasis and poor prognosis in patients with resectable pancreatic cancer [16]. Our results indicate that in LN16 positive patients, the preoperative serum CA125 levels predict prognosis after surgical resection. ROC analysis demonstrated that the area under ROC curve was 0.675, when the optimal CA125 cut off value for LN-16 positive patients was 18.62 U/ml, with a sensitivity of 70.0% and specificity 75.0% respectively. These results indicate that when the preoperative CA125 level is lower than 18.62 U/ml, the patient will benefit from the surgery, even with para-aortic lymph node metastasis (Figure 4).

### JAK2 signaling pathway may be involved in the mechanism underlying the different surgical outcomes in LN16-involved patients

**Figure 5:**
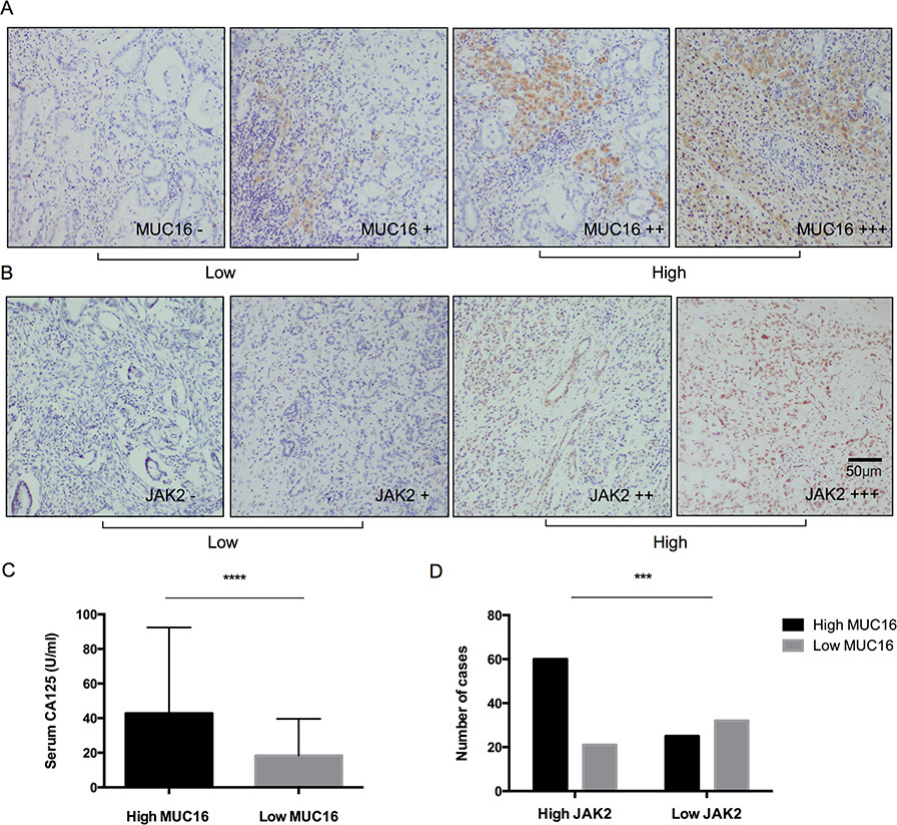
Expressions of mucin-16 (MUC16) and JAK2 in pancreatic cancer tissues from patients with LN16 metastasis (**A, B**) The expression levels of MUC16 and JAK2 were classified into low (−, +: score 0-1) and high (++, +++: score 2–3) groups according to the scores from IHS staining (original magnification ×200). Scale bar, 50 μm. (**C**) The expression of MUC16 in pancreatic cancer tissues was consistent with the preoperative serum CA125 levels in patients with LN16 metastasis (*p* < 0.001). (**D**) Expression of JAK2 correlated with MUC16 levels in pancreatic cancer patients with LN16 metastasis (*p* < 0.001).

To uncover the mechanism by which the preoperative serum CA125 level can distinguish the “surgical benefit” group of patients with pancreatic head cancer with LN16 metastasis, the expression of CA125 (mucin-16) and its related protein JAK2 was analyzed in primary pancreatic cancer tissues using immunohistochemistry assay. We found that in pancreatic cancer tissues, the expression of JAK2 correlated with the expression of mucin-16, which was associated with the preoperative serum CA125 levels of patients with pancreatic head cancer with LN16 involvement (Figure 5).

## DISCUSSION

According to the pathological staging system for pancreatic cancer proposed by the Japanese Pancreas Society (Supplementary Figure S1), para-aortic lymph nodes are attributed to Group 3, and para-aortic lymph node involvement is characterized as distant metastasis [17]. Several researchers investigated the lymphatic drainage pattern from the head of the pancreas to the para-aortic lymph nodes, and determined that the lymph nodes around the superior mesenteric artery (SMA) should provide critical access to the para-aortic lymph nodes [12, 18]. However, a more recent study used an imaging agent to observe the lymphatic flow pathway for pancreatic head cancer, and found that the incidence of LN16 metastatic involvement was significantly higher than the incidence of LN14 involvement. The time required for the imaging agent to arrive at the para-aortic region was less than was required to arrive at the SMA lymph nodes [19]. To address this discrepancy, Japanese researchers indicated that there could be different lymphatic metastatic pattern based on different tumor locations in the head of pancreas, including the ventral and dorsal pancreas. If the tumor occupies both parts of the pancreas, the lymphatic metastatic profile will be more complicated [20].

In the present study, we found that the rate of para-aortic lymph node involvement in tumors confined to the pancreatic uncinate process was significantly higher than that of tumors derived from other domains of the head of pancreas. Metastasis to Group 1 or 2 lymph nodes did not correlate with LN16 metastasis in patients with uncinate process disease, although there was a statistically significant difference between Group 1 and 2 lymph nodes and LN16 metastasis in the overall sample. The results indicate that in tumors from the uncinate process of the pancreas, either discontinuous or “skip” metastasis may often occur, or LN16 may not be a Group 3 lymph node station. In our survival analysis, smaller tumor size and a non-uncinate process tumor location were associated with poor prognosis after surgical resection in LN16-positive patients, further indicating an individual metastatic pattern of para-aortic lymph nodes based on tumor location. Despite the lack of level I evidence, it should not be automatically assumed that para-aortic lymph nodes belong to Group 3 in every resectable case and, consequently, should not be recommended for curative surgery regardless of tumor location, especially in the case of tumors located at the uncinate process of the pancreas.

Pancreatic cancer tends to metastasize at early stages, and metastatic involvement of the peri-pancreas lymph nodes has always been considered a critical prognostic value for this lethal disease [3]. To find a reliable indicator to evaluate prognosis after curative surgery, several variables, including numbers of positive lymph nodes (PLN), lymph node ratio (LNR), and total number of examined lymph nodes (ELN), were assessed for their ability to predict disease outcome [21]. Although there are different opinions regarding which single variable is the best prognostic marker [3, 22], our previous study indicated that an LNR ≥ 0.4 was a crucial factor with which to identify patients with potential poorer survival outcomes after surgery [14]. In the current LN16-positive cohort, LNR < 0.25 indicated a longer survival time after a curative surgical resection, that is, not all the cases with LN16 involvement were destined to experience a bad outcome after radical surgery. The results suggest that “heterogeneity” may underlie metastasis to the para-aortic lymph nodes in patients with pancreatic head cancer.

Changes in tumor burden are important for evaluation of pancreatic cancer responses to therapeutics [23, 24]. Earlier, we reported that two measures of metabolic tumor burden, metabolic tumor volume (MTV) and total lesion glycolysis (TLG), can be used to predict overall survival and recurrence-free survival for patients with pancreatic cancer after radical dissection [23]. In addition, high serum CA19-9 levels, which represent serum tumor burden, often indicate occult metastasis or a poor disease outcome for resectable pancreatic cancer [24]. In this study, the presence of para-aortic lymph node metastasis was associated with high serum CA19-9 levels and involved lymph nodes, rather than large tumor size. Although LN16 may not be attributed to a distant lymph node station for some cases, such as tumors from the dorsal pancreas or the uncinate process of the pancreas, due to a unique anatomical position adjacent to the inferior vena cava, which is an area abundant in retroperitoneal lymphoid tissues, vessels, and nerve fibers, systemic metastasis may develop in early stages of the disease.

Since the presence of para-aortic lymph node metastasis suggested occult distant metastasis, we investigated whether curative surgery is necessary for LN16-positive patients. We found that there was no difference in overall survival between patients with resected tumors with involved para-aortic lymph nodes and patients with unresectable, locally advanced, but chemo-responsive disease. Interestingly, the outcome was different when we separated the LN16-positive patients into two subgroups based on preoperative serum CA125 levels. CA125, a gynecological tumor-associated biomarker that is also known as mucin-16, plays a critical role in metastatic invasion and chemotherapy resistance [25]. Mucin-16 has been recently shown to be an effective complement to CA19-9 in the early detection of pancreatic cancer [26]. We have previously reported that a high level of CA125 may indicate unresectable pancreatic cancer, even when initially judged as resectable by a preoperative enhanced CT scan, and that the predictive value determined by ROC curve is even better than the classic CA19-9 [15]. Our study group also found that preoperative serum CA125, CA19-9, and CEA levels could determine the cohort of patients with poor outcomes, even after curative resection [24]. More recently, high preoperative CA125 level was identified to be an independent risk predictor for overall survival (OS) and recurrence-free survival (RFS) in patients with resected pancreatic cancer [16].

In this study, we have found that although positive LN16 indicates an overall poor survival outcome, the subgroup of patients with CA125 < 18.62 U/ml might still benefit from surgery. This intriguing result suggests that even though LN16 is involved, the primary tumor may be still resectable. Emerging research has suggested that the tumor promoting and metastatic activity of mucin-16 (CA125) is involved in the JAK2 signaling pathway [25, 27]. In the present study, preoperative serum CA125 level and mucin-16 expression in primary tumor tissues correlated with the nuclear levels of JAK2 protein, rather than its downstream effectors STAT3 or pSTAT3 (data not shown). This finding may shed light on the function of CA125 in this unique cohort of patients with pancreatic cancer.

In summary, para-aortic lymph nodes should not be automatically categorized as Group 3 or distant lymph node stations, regardless of dorsal or ventral tumor location in the pancreas. Further research is needed. Although LN16 involvement indicates a poor overall prognosis, metastasis to LN16 could lead to different surgical outcomes, depending on the preoperative serum CA125 levels. Consequently, LN16 involvement in resectable pancreatic head cancer may indicate a different prognosis for different individuals. Pancreatic surgeons must make every effort to discriminate the cohort of patients that will still benefit from surgery, even with an involved para-aortic lymph node, rather than completely abandoning the surgical opportunity that might be the final opportunity for long-term survival for patients with pancreatic cancer.

## MATERIALS AND METHODS

### Study design

This study included 579 patients with pancreatic head ductal carcinoma who underwent radical pancreatectomy with extended lymphadenectomy, including para-aortic lymph nodes, at Fudan University Shanghai Cancer Center and Huashan Hospital between February 1999 and February 2014. Another cohort of 140 patients identified to be unresectable due to extensive local advancement but chemo-responsive was also included. All surgically resected cases were pathologically diagnosed as invasive pancreatic ductal adenocarcinomas, including 336 males and 243 females, with a mean age of 61.56 years (range 30–81 years). A pathological staging system for pancreatic cancer proposed by Japanese Pancreas Society (JPS, 2010) was used to evaluate the pathological findings. According to the JPS staging system, regional lymph nodes of pancreatic head cancer are divided into two groups, Group 1 (LN13, 17) and Group 2 (LN6, 8, 12, 14). Group 3 contains distal lymph nodes, such as para-aortic stations that are usually attributed to distant metastasis. All patients with resectable disease underwent an extended lymphadenectomy that included Group 1, Group 2, and para-aortic lymph nodes. The patients with unresectable disease were pathologically confirmed as having pancreatic cancer by an endoscopic ultrasound-guided fine-needle aspiration. Most received gemcitabine-based chemotherapy. Lymph node ratio (LNR) was calculated by dividing the number of metastatic lymph nodes by the total number of examined lymph nodes.

### Follow-up

Overall survival is the primary end-point of this study. Both groups of patients were followed up until death or February 2016. Survival time was calculated from the date of final diagnosis to the date of the last follow-up or death. The study was approved by the Ethics Committee of the Fudan University Shanghai Cancer Center and Huashan Hospital. Written informed consent was obtained from all of the subjects enrolled in this study.

### Measurements of MUC16 and JAK2 levels in pancreatic cancer tissues

Immunohistochemistry (IHC) staining was performed as previously described [28]. Two pathologists assessed the cytoplasmic expression of MUC16 (Abcam, 1:100) and nuclear expression of JAK2 (Abcam, 1:500), based on the staining percentage and intensity. MUC16 and JAK2 expressions were categorized into - (score 0), + (score 1), ++ (score 2), and +++ (score 3).

### Statistical analysis

Statistical analysis was performed with SPSS software (version 22.0, IBM). The measurement data were expressed as mean ± standard deviation, while categorical data were expressed as rates. Student's *t* test and non-parametric Mann-Whitney test were used for continuous variables. Categorical variables were compared using χ^2^ test. The prognostic value of clinicopathologic factors was evaluated by univariate analysis among the LN16 positive patients. Variables found to be significant by univariate analysis were subjected to multivariate analysis with a Cox proportional hazards model. Survival rates for each variable were evaluated by Kaplan-Meier curves using Log-rank or Breslow tests. Receiver operating characteristic (ROC) analysis was performed and the area under the curve (AUC) value was calculated to determine a CA125 or LNR cut off value. A two-tailed *p*-value of less than 0.05 was considered statistically significant.

## SUPPLEMENTARY MATERIALS FIGURE


