# Supplementary material for: Which patients with para-aortic lymph node (LN16) metastasis will truly benefit from curative pancreaticoduodenectomy for pancreatic head cancer?
Source: Oncotarget. 2016 Apr 11;7(20):29177–86. doi: 10.18632/oncotarget.8690 (PMC5045387; doi:10.18632/oncotarget.8690)
Supplement: Supplementary file 1 [file oncotarget-07-29177-s001.pdf]

Which patients with para-aortic lymph node (LN16) metastasis will truly benefit from curative pancreaticoduodenectomy for pancreatic head cancer?

Supplementary Materials

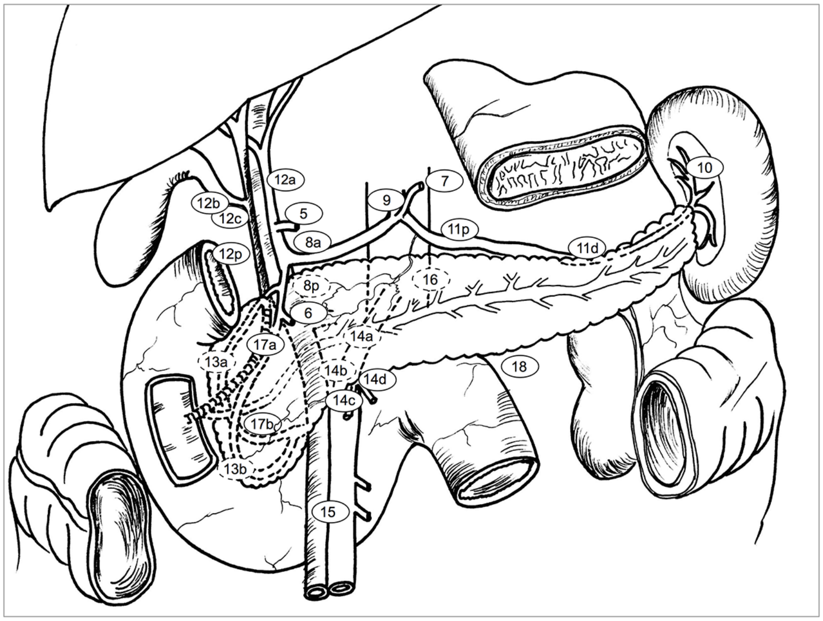

Supplementary Figure S1: Nomenclature of peri-pancreatic lymph nodes stations (adapted from the second English edition of the Classification of Pancreatic Carcinoma proposed by Japanese Pancreas Society). Lymph node stations: No. 5, suprapyloric lymph nodes; No. 6, infrapyloric lymph nodes; No. 7, lymph nodes along the left gastric artery; No. 8a, lymph nodes in the anterosuperior group along the common hepatic artery; No. 8p, lymph nodes in the posterior group along the common hepatic artery; No. 9, lymph nodes around the celiac artery; No. 10, lymph nodes at the splenic hilum; No. 11p, lymph nodes along the proximal splenic artery; No. 11d, lymph nodes along the distal splenic artery; No. 12a, lymph nodes along the hepatic artery; No. 12p, lymph nodes along the portal vein; No. 12b, lymph nodes along the bile duct; No. 12c (located next to 12b), lymph nodes around the cystic duct; No. 13a, lymph nodes on the posterior aspect of the superior portion of the head of the pancreas; No. 13b, lymph nodes on the posterior aspect of the inferior portion of the head of the pancreas; No. 14a, lymph nodes at the origin of superior mesenteric artery; No. 14b, lymph nodes on the right side of superior mesenteric artery; No. 14c, lymph nodes in the anterior group of superior mesenteric artery at middle colic artery; No. 14d, lymph nodes on the left side of superior mesenteric artery; No. 15, lymph nodes along the middle colic artery; No. 16, lymph nodes around the abdominal aorta; No. 17a, lymph nodes on the anterior surface of the superior portion of the head of the pancreas; No. 17b, lymph nodes on the anterior surface of the inferior portion of the head of the pancreas; and No. 18, lymph nodes along the inferior margin of the pancreas.
